# Virtual fitness buddy ecosystem: a mixed reality precision health physical activity intervention for children

**DOI:** 10.1038/s41746-024-01133-5

**Published:** 2024-05-21

**Authors:** Sun Joo (Grace) Ahn, Michael D. Schmidt, Allan D. Tate, Stephen Rathbun, James J. Annesi, Lindsay Hahn, Eric Novotny, Christian Okitondo, Rebecca N. Grimsley, Kyle Johnsen

**Affiliations:** 1 Grady College of Journalism and Mass Communication, University of Georgia, Athens, GA USA; 2 Department of Kinesiology, University of Georgia, Athens, GA USA; 3 Epidemiology and Biostatistics, College of Public Health, University of Georgia, Athens, GA USA; 4 Kinesiology Department, California State University, Monterey Bay, Seaside, CA USA; 5 Department of Communication, University at Buffalo, Buffalo, NY USA; 6 Workplace Research and Insights, Haworth, Inc., Holland, MI 49424 USA; 7 Center for Advanced Computer-Human Ecosystems, University of Georgia, Athens, GA USA; 8 College of Engineering, University of Georgia, Athens, GA USA

**Keywords:** Randomized controlled trials, Communication

## Abstract

6–11-year-old children provide a critical window for physical activity (PA) interventions. The Virtual Fitness Buddy ecosystem is a precision health PA intervention for children integrating mixed reality technology to connect people and devices. A cluster randomized, controlled trial was conducted across 19 afterschool sites over two 6-month cohorts to test its efficacy in increasing PA and decreasing sedentary behavior. In the treatment group, a custom virtual dog via a mixed reality kiosk helped children set PA goals while sharing progress with parents to receive feedback and support. Children in the control group set PA goals using a computer without support from the virtual dog or parents. 303 children had 8+ hours of PA data on at least one day of each of the 3 intervention time intervals. Conversion of sedentary time was primarily to light-intensity PA and was strongest for children with low baseline moderate-to-vigorous PA than children above 45 min of baseline moderate-to-vigorous PA. Findings suggest that the VFB ecosystem can promote sustainable PA in children and may be rapidly diffused for widespread public health impact.

## Introduction

Young to middle childhood (6–11 years old) is a critical time to build healthy lifestyles that can be sustained into adulthood. Engaging in adequate levels of moderate-to-vigorous intensity physical activity (PA) is a widely recommended health behavior, as childhood PA has been linked to resilience against health problems later in life, such as obesity and related chronic diseases^1,2^ and premature mortality^3,4^. The majority of youth fail to get the recommended amount of PA, with older children in middle and high schools experiencing a precipitous decline in PA compared to younger children, in part due to longer time spent in classrooms engaged in sedentary activities^5–7^. Because interventions are far less successful once obesity is established in childhood^8^, children between the ages of 6 and 11 in elementary school provide a critical window for PA interventions to motivate and sustain healthy PA habits and reduce sedentary behavior^9^.

Numerous health interventions that leverage emerging digital technologies have been developed in recent years, such as exergames (digital games that require players to perform PA or demonstrate their knowledge of the benefits of PA), in efforts to encourage children to associate PA with the fun, game-like feel of digital games^10–13^. Although these technology-mediated PA interventions have demonstrated considerable promise, they often yield modest effects, with short-lived and transient PA increases following the initial intervention^14–17^.

Self-determination theory, a social psychological framework for predicting and describing human motivation for behavior change^18^, provides one explanation for this outcome: when children associate PA behaviors with extrinsic sources of motivation (e.g., points, badges), PA behaviors can stop when the extrinsic rewards end^19^. These findings suggest that linking PA to the technology interface alone (e.g., emphasizing extrinsic rewards in an exergame) deprives children of the opportunity to become the change agent to internalize and integrate PA into their lives, diminishing the overall potential of technology-mediated health interventions for longer-term behavior change.

Self-determination theory contends that there are basic, universal needs that motivate human behaviors at all stages of life through the inherent satisfaction and pleasure derived from the autonomy, competence, and relatedness that stem from these activities^20^. When these universal psychological needs are met, individuals become *intrinsically* motivated to set and meet self-determined goals that build sustained and internalized behavior change^21^. To apply these theoretical tenets to the domain of children’s PA, health interventions should aim to build: (1) autonomy (e.g., encouraging children to set their own PA goals), (2) competence (e.g., tailoring the intervention to each child’s PA ability to encourage perceived competence^22^) and (3) relatedness (e.g., opportunity to communicate and feel connected with peers and parents).

Emerging digital technologies afford novel features that allow researchers and practitioners to precisely tailor the intervention to each child based on individually generated data and enhance social connections to their parents and peers in scalable and cost-effective ways that were difficult in traditional health interventions^23^. The precision health approach allows individuals to set the pace of their own health intervention and serve as vehicles of change, increasing the likelihood of internalizing behavior change^24^. Guided by self-determination theory, we developed and evaluated a community-level precision health intervention for children that integrates the theoretical tenets into a scalable and cost-effective PA program to increase and sustain PA in children through unstructured play—the virtual fitness buddy (VFB) ecosystem.

The VFB ecosystem encourages children to engage with a custom virtual agent (an algorithm-driven digital dog) to set and meet self-determined PA goals while sharing progress with family and peers in real-time and enveloping each child with feedback and social support. The virtual dog encourages children to autonomously determine their PA goal for the week without externally set goals to nurture perceived autonomy for PA^25,26^. This virtual dog is designed to assist children in setting and meeting PA goals via a mixed reality kiosk with built-in sensor-based technologies; children can experience mastering PA goals and setting new ones as they experience their PA in the real world, blending with vicarious reinforcement in the virtual world through their dog, leading to heightened perceived competence for PA^27^. The virtual dog demonstrates vicarious reinforcement by becoming slimmer in appearance, faster in its actions, and more energetic in its interactions based on children’s PA achievements tracked by wearable activity monitors (i.e., a Fitbit); the more PA goals children achieve, the more energy the virtual dog gains, allowing children to interact with the virtual dog for longer periods of time^28^.

**Fig. 1:**
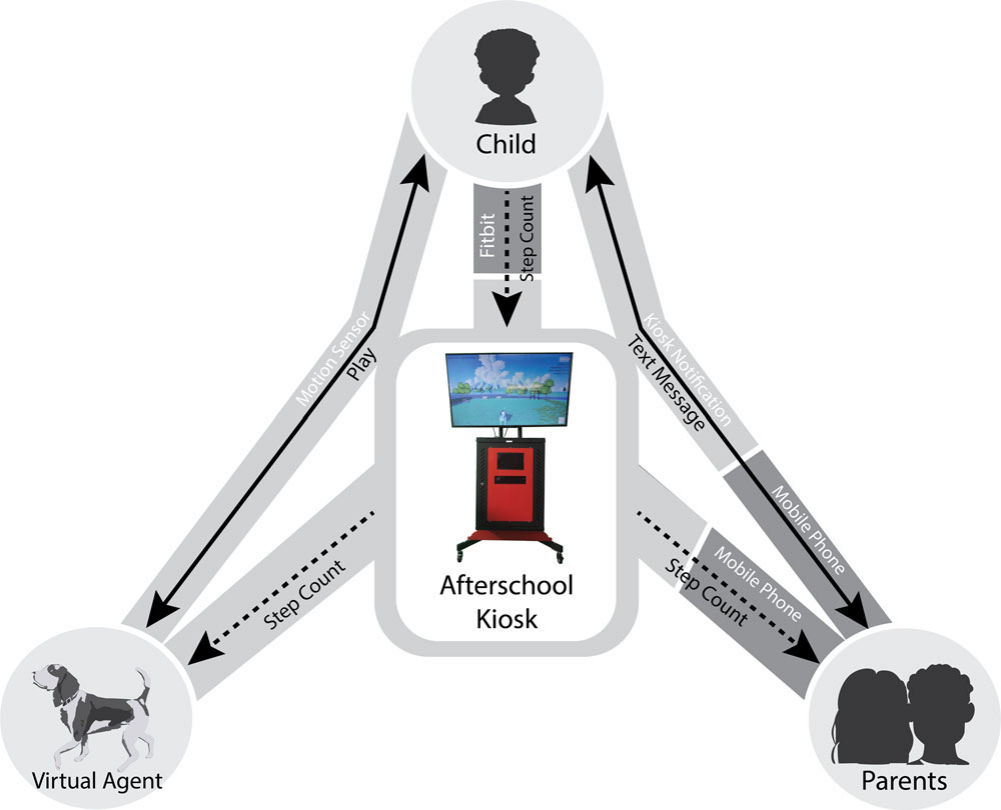
Data flow within the virtual fitness buddy ecosystem. The mixed reality kiosk served as the central communication hub between parents and children. All children wore Fitbits on the nondominant wrist, and their step count was synced whenever they visited their virtual dog through the kiosk. The virtual dog’s appearance and behavior changed to reflect the PA performed by the child; more PA led to a slimmer virtual dog with a happier demeanor. Each time the child visited the kiosk and interacted with the dog, their PA performance synced to the kiosk was reported to the child’s parents via text message.

The motion-tracking mixed reality kiosk served as a central point of communication within the ecosystem. Based on automated input from children’s wearable activity monitors, the virtual dog provided precise, tailored feedback based on each child’s PA progress. If the child met the self-set goal, the virtual dog praised the child and asked him or her to play via the motion-tracking mixed-reality kiosk through voice, hand gestures, and body movement, mimicking human-pet interactions in the real world. If the child’s PA progress fell short of the self-determined goal, the virtual dog offered accurate feedback and supportive comments to encourage further PA. When children interacted with the virtual dog, the kiosk automatically sent notifications to parents regarding PA progress so that parents could also send encouraging messages to their children, which were then delivered through the kiosk. Therefore, the VFB ecosystem allowed children to receive complementary, individually tailored social support from both their parents and the virtual dog to foster perceived relatedness and increase program adherence during the intervention and internalization of the health behavior change (Fig. 1)^29,30^.

The current study reports the results from a randomized controlled clinical trial in collaboration with the YMCA of Metropolitan Atlanta. We tested the efficacy of the VFB ecosystem across two cohorts of parent–child dyads recruited from 19 elementary schools and YMCA branches in the metro Atlanta, Georgia area, each cohort lasting for six months during the academic year. Specifically, we compared the VFB ecosystem’s efficacy in increasing PA among 6–11-year-old children against an active control group, which employed a computer system to assist children in setting and meeting PA goals but without social support or feedback from the virtual dog or their parents. Our sampling targeted a wide and diverse range of communities with respect to SES and demographic factors, including 12 locations in cohort 1 and seven in cohort 2.

Beyond its scale, this study is unique in that we were able to track the real-time progress of 303 children’s PA over six months across 19 different study sites. Children and their participating family members were equipped with Fitbit devices for children to engage with their custom virtual dog via the mixed reality kiosk based on their PA performance for the duration of the study, as well as Actigraphs (research-grade PA tracking device) for 1-week periods at three time-points throughout the trial (baseline, 3-months, and 6-months). The rich data derived from these devices allowed the research team to observe patterns in children’s light, moderate, and vigorous-intensity PA, as well as sedentary behavior, over 6 months during the intervention.

## Results

### Study population

Of the 303 primary caregivers, three-quarters were female, and parent body mass index (BMI) was 27.3 ± 7.3 (Treatment; T) and 27.9 ± 5.6 (Control; C), indicating that caregivers were overweight on average (Table 1). The 303 children reported in the sample were majority boys, and the age and sex compositions were approximately the same in treatment and control arms (8.1 ± 1.4 years old in T, 8.1 ± 1.4 years old in C). Child baseline age-/sex-adjusted BMI percentile was 68.9 ± 26.9 (T) and 68.7 ± 27.9 (C), and approximately 42% (T) and 38% (C) of children had BMI percentile greater than 85 indicating moderate prevalence of child overweight in the sample. The socio-demographic composition of the school-based randomization to treatment and control conditions achieved overall balance in the two comparison arms.

**Table 1:** Baseline demographic characteristics of participant parents and children assigned to experimental conditions (*n* = 19 schools; *n* = 303 parent/child dyads)

	Condition	
	Treatment	Control
Individual characteristics	*n* (%)	*n* (%)
Parent female	118 (77.6%)	114 (75.5%)
Child female	62 (40.8%)	66 (43.7%)
Child age ± sd	8.1 ± 1.4	8.1 ± 1.4
Parent BMI ± sd	27.3 ± 7.3	27.9 ± 5.6
Child BMI%ile ± sd	68.9 ± 26.9	68.7 ± 27.9
Child overweight	64 (42.1%)	58 (38.4%)
*Child race/ethnicity*		
Hispanic	4 (2.6%)	8 (5.3%)
White	63 (41.5%)	56 (37.1%)
Black	52 (34.2%)	41 (27.2%)
East Asian	5 (3.3%)	5 (3.3%)
South Asian	13 (8.6%)	16 (10.6%)
Other	2 (1.3%)	4 (2.7%)
Multi-racial	9 (5.9%)	15 (9.9%)
Prefer not to report	4 (2.6%)	6 (4%)
Family characteristics		
*Household income*		
<$30,000	20 (13.2%)	11 (7.3%)
$30,000–$59,999	16 (10.5%)	17 (11.3%)
$60,000–$99,999	21 (13.8%)	28 (18.5%)
$100,000+	71 (46.7%)	76 (50.3%)
Prefer not to report	24 (15.8%)	19 (12.6%)
*Parent education*		
HS/GED	10 (6.8%)	10 (6.9%)
Some college	20 (13.6%)	12 (8.3%)
Associates/trade/vocational	14 (9.5%)	15 (10.3%)
Bachelors	55 (37.4%)	54 (37.2%)
Masters	37 (25.2%)	43 (29.7%)
Doctoral/terminal degree	11 (7.5%)	11 (7.6%)
Prefer not to report	5 (3.3%)	6 (4%)
*Parent relationship status*		
Married	92 (60.5%)	102 (67.6%)
Divorced/separated/widowed	21 (13.8%)	19 (12.6%)
Single/never married	28 (18.4%)	17 (11.3%)
Cohabitating	3 (2%)	5 (3.3%)
Prefer not to report	8 (5.3%)	8 (5.3%)

### Intent-to-treat treatment effects of the VFB ecosystem

#### Moderate-vigorous intensity physical activity (MVPA)

MVPA was examined longitudinally over three measurement points during the intervention and compared between children attending schools randomly assigned to the intervention (T) or the control condition (C). The child-level intraclass-correlation coefficient (ICC) was 0.204 over the study period, reflecting a small-to-moderate correlation in daily MVPA within children, and school-level ICC was effectively zero indicating dissimilar patterns of MVPA in the student populations at schools.

**Fig. 2:**
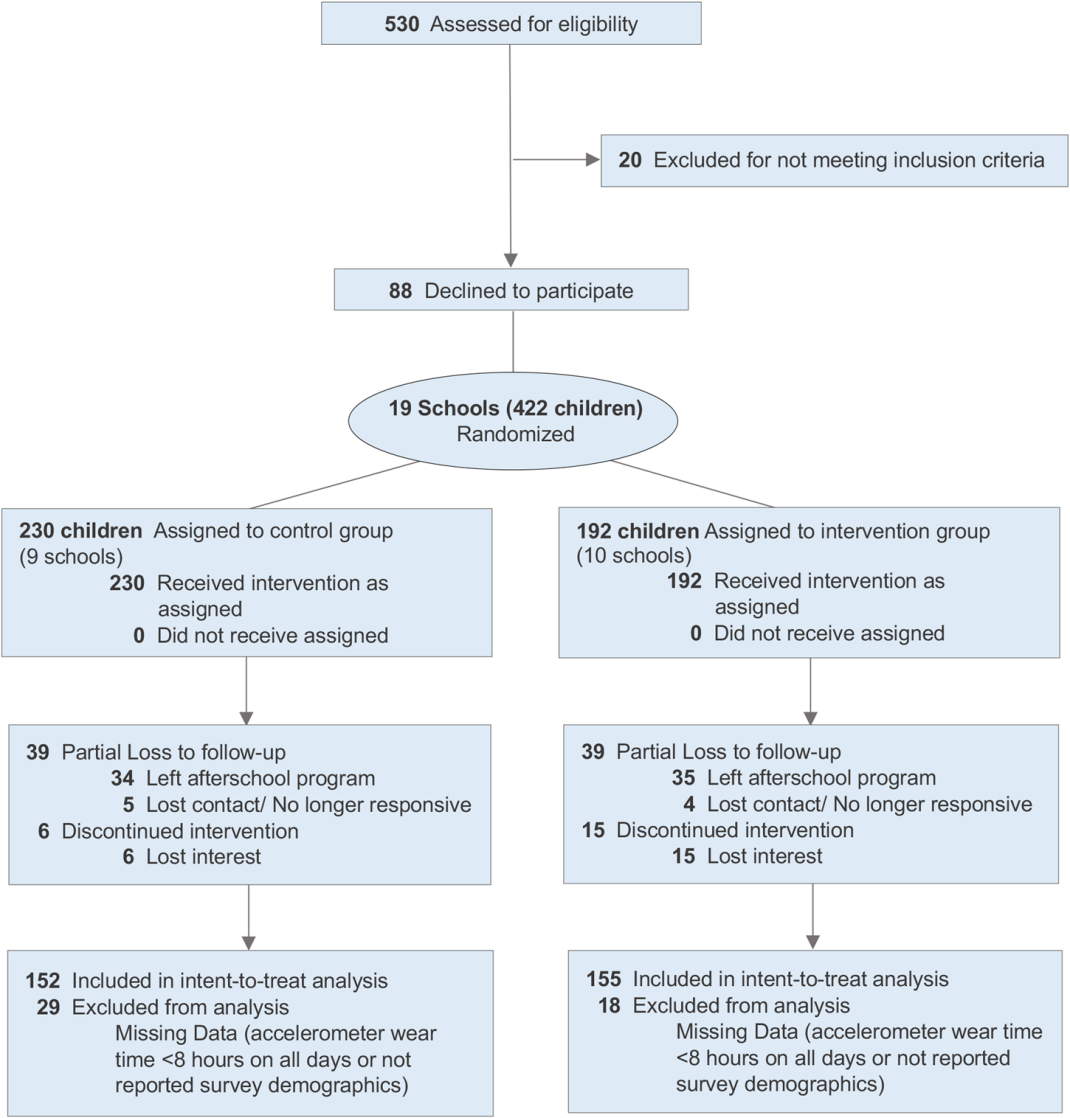
CONSORT flowchart of participating children in the VFB ecosystem clinical trial. For this multisite, cluster-randomized, controlled trial, a total of 422 children participated across 19 afterschool sites affiliated with the YMCA of Metropolitan Atlanta. After accounting for attrition and exclusions from data analysis, 152 children were included in the intent-to-treat analysis for the control group, and 155 children were included in the intent-to-treat analysis for the treatment group.

Results (Table 2) indicated that treatment effects at baseline Time 1 (MVPA minutes −3.8, 95% CI: −8.5, 0.9), 3 months later at Time 2 (MVPA minutes 3.9, 95% CI: −2.5, 10.2), and 6 months later at Time 3 (MVPA minutes −6.0, 95% CI: −14.5, 2.4) were not different between the two arms at *p* < 0.05. Standardized treatment effect sizes (Cohen’s *d*) are presented visually for all PA and sedentary behavior outcomes (Fig. 2). The overall intent-to-treat (ITT) test of treatment interaction effects over time did not support that the intervention had favorable or unfavorable effects on the child’s MVPA (*p* = 0.092). Within-arm examination of MVPA levels indicated that at Time 1, both arms exhibited levels of MVPA comparable to the U.S. guidelines of 60 min per day (T: 53.9 min; C: 57.7 min). In the treatment group, MVPA improved by about 3.5 (95% CI: −2.29, 9.35) minutes between Time 1 and Time 2, but declined by 4.2 (95% CI: −10.2, 1.9) minutes on average in the control condition (a post hoc statistical test was not conducted).

**Table 2:** Treatment-control predicted marginal accelerometer physical activity and sedentary time minutes and treatment effects over time (*n* = 303 children; *n* = 19 schools)

	Time 1	Time 2	Time 3	Overall interaction *P* value
*Moderate-vigorous intensity PA*				
Treatment arm, adjusted mean (95% CI)	53.9 (50.6, 57.2)	57.4 (52.3, 62.6)	51.5 (46.4, 56.6)	
Control arm, adjusted mean (95% CI)	57.7 (54.6, 60.8)	53.5 (49.8, 57.3)	57.5 (50.9, 64.1)	
Treatment effect (contrast and 95% CI)	−3.8 (−8.5, 0.9)	3.9 (-2.5, 10.2)	−6 (−14.5, 2.4)	0.092
*Vigorous intensity PA*				
Treatment arm, adjusted mean (95% CI)	18.4 (17.1, 19.7)	20.3 (17.6, 22.9)	18.2 (15.9, 20.6)	
Control arm, adjusted mean (95% CI)	20.4 (18.8, 21.9)	18.9 (16.9, 20.9)	21.3 (18.3, 24.2)	
Treatment effect (contrast and 95% CI)	−2 (−4.1, 0.2)	1.3 (-2, 4.7)	−3 (−6.8, 0.8)	0.150
*Moderate intensity PA*				
Treatment arm, adjusted mean (95% CI)	35.2 (33, 37.3)	36.8 (34.2, 39.5)	32.9 (30, 35.9)	
Control arm, adjusted mean (95% CI)	37.1 (35.6, 38.6)	34.4 (32.5, 36.4)	36 (32.1, 39.9)	
Treatment effect (contrast and 95% CI)	−1.9 (−4.6, 0.8)	2.4 (-0.8, 5.7)	−3.1 (−8.1, 1.9)	0.071
*Light intensity PA*				
Treatment arm, adjusted mean (95% CI)	246.6 (235.7, 257.6)	246.4 (237.9, 254.9)	229.6 (216.4, 242.7)	
Control arm, adjusted mean (95% CI)	249.2 (241.2, 257.2)	229 (225, 233)	227 (214.6, 239.5)	
Treatment effect (contrast and 95% CI)	−2.6 (−16.7, 11.5)	17.4 (7.7, 27.2)	2.5 (−16.4, 21.5)	**0.001**
*Daily steps*				
Treatment arm, adjusted mean (95% CI)	8679 (8098, 9259)	8608 (7914, 9303)	7955 (7208, 8702)	
Control arm, adjusted mean (95% CI)	8921 (8474, 9367)	8325 (7810, 8841)	8585 (7824, 9346)	
Treatment effect (contrast and 95% CI)	−242 (−1008, 524)	283 (−577, 1143)	−630 (−1724, 464)	0.17
*Sedentary time*				
Treatment arm, adjusted mean (95% CI)	487.1 (474, 500.2)	483.6 (472.6, 494.5)	506.5 (489.3, 523.7)	
Control arm, adjusted mean (95% CI)	481 (471.6, 490.3)	505.2 (499.9, 510.6)	503.1 (485.5, 520.6)	
Treatment effect (contrast and 95% CI)	6.2 (−10.5, 22.9)	−21.7 (−34.1, −9.2)	3.4 (−22.1, 29)	**0.005**

Models adjusted for hours of accelerometer wear time and restricted to daily wear time of 8+ hours; child overweight, sex, age, and race; parent overweight, sex, and educational attainment.

Interpretation example: Child physical activity and sedentary time measured by accelerometer were measured longitudinally over three observation periods. Multilevel mixed models with random intercepts for school and child and a random slope for observation day within the child were fitted to examine treatment effects for a virtual pet intervention on child moderate-to-vigorous intensity physical activity (MVPA). The interaction between the treatment/control group and longitudinal period was not statistically different for the two randomized treatment groups performed at the school level (interaction *p*-value = 0.092). Plots of the slope of predicted MVPA indicated increases within the treatment arm by period 2 that were not sustained by period 3. The pattern was reversed for the control group indicating a decline in MVPA of about 4.2 min between periods 1 and 2 and a return to the original level of MVPA by period 3. In period 2, the MVPA treatment effect was estimated to be 3.9 more minutes of MVPA in the treatment group compared to the control group, but the increase was not statistically significant (95% CI: −2.5, 10.2). The period 2 treatment effect is characterized by an increase in MVPA among the treatment group and a decline in MVPA among the control group between baseline and first follow-up.

#### Daily composition of sedentary behavior

Sedentary behavior was examined to determine whether daily minutes of sedentary time converted to PA in the light, moderate, and vigorous intensity categories, reflecting an overall healthier composition of daily PA and sedentary behavior. Child-level ICC was 0.181 over the study period, indicating a small-moderate correlation in daily sedentary behavior within children, and school-level ICC was 0.004, indicating dissimilar patterns of sedentary behavior in the student populations. At Time 1, sedentary minutes were 487.1 or 8.1 h (T) and 481 or 8.0 h (C), reflecting similar levels of sedentary time in the two arms (ITT contrast: 6.2 min, 95% CI: −10.5, 22.9). A favorable decline in sedentary time at Time 2 was exhibited in the treatment compared to the control group of −21.7 sedentary minutes (95% CI: −34.1, −9.2), which was statistically significant at *p* < 0.001. Overall, there was statistical evidence that the pattern of sedentary time was different in the treatment arm compared to the control group at *p* = 0.005, but the strongly favorable intervention effect at Time 2 was not maintained at Time 3.

### Post hoc stratified sensitivity analyses of treatment effects

A post hoc analysis was conducted to examine whether intervention effects were stronger for children further from the U.S. physical activity guidelines of 60 min per day of MVPA at baseline Time 1. One-third of the analytic sample (*n* = 99 children) had a baseline daily MVPA of below 45 min, and these children were evenly distributed across the treatment and control arms (*n* = 50 children in the treatment arm). We observed the same patterns of treatment effects being strongest at Time 2; however, there was descriptive evidence that the conversion of sedentary behavior to PA was strongest for children with a baseline MVPA below 45 min (Fig. 2). In children with lower MVPA, treatment effects were −37.3 min SB/day at Time 2 compared to Time 1, but the same effect was −23.9 min sedentary behavior/day among children with higher baseline MVPA. These results indicated that the treatment effects on reducing sedentary behavior may be about 55% stronger among children with lower MVPA compared with children who have higher MVPA. Notably, 80% of the sedentary behavior reduction was converted to light-intensity PA for children with lower MVPA, while 66% of the sedentary behavior reduction was converted to light PA for children with higher MVPA. This descriptive difference in the conversion of sedentary behavior indicated that the intervention may have improved engagement in light-intensity PA for children with lower MVPA and may have improved engagement in moderate and vigorous-intensity PA at a slightly higher rate for more physically active children.

Current pet ownership was also evaluated as a potential synergistic factor in enhancing treatment effects. About one-third of the sample (*n* = 106) reported owning a dog, and almost two-thirds of dog owners were white (*n* = 67). At first follow-up, both groups of dog owners exhibited increased conversion of sedentary time to light intensity PA (interaction *p* values < 0.05), however, the treatment effect was more than twice as strong for dog owners (light intensity PA effect: +26.6 min) compared with non-dog owners (light intensity PA effect: +11.5 min). Further, this treatment effect was sustained and slightly increased for children with dogs during the second follow-up period (light intensity PA effect: +30.5 min) but not for children without dogs (light intensity PA effect: −11.5 min).

### Post hoc stratified sensitivity analyses of implementation effects

A sensitivity analysis was conducted to examine the effectiveness of system enhancements made to the VFB ecosystem kiosk between cohorts 1 and 2 that aimed to increase intervention fidelity and intervention uptake. There were improvements in treatment effects for cohort 2 compared to cohort 1, suggesting successful deployment of intervention modifications for cohort 2. For example, the pattern of treatment effects between Time 1 to Time 2 strengthened for cohort 2 as indicated by the 7.6 MVPA minute per day gain in the treatment arm compared to the control arm (95% CI: 3.1, 12.0). This effect was comparatively smaller in cohort 1 (2.1 MVPA minute per day gain, 95% CI: −8.3, 12.6). Cohort 2 also exhibited a clinically meaningful population-average improvement in MVPA minutes between Time 1 and Time 2, with daily PA above U.S. guidelines of 60 MVPA minutes at Time 2 (61.8 min, 95% CI: 60.1, 63.6). Light intensity PA consistently exhibited the strongest PA compositional gains compared to other levels of PA intensity, and sedentary time was reduced by about a half hour in the treatment arm compared to the control arm at Time 2. As with the primary ITT analysis, intervention gains were not sustained at Time 3 and reflect an overall return to baseline levels of PA and SB.

Direct kiosk interactions with the virtual pet (i.e., time durations when the child was actively playing with the pet) were examined for both cohorts to evaluate how implementation differed across school sites. Table 3 presents descriptive statistics that characterize the frequency and duration of child interaction with the kiosk. During the intervention period, the proportion of school days with school-level average playtime >30 s, >100 s, and >150 s are presented.

**Table 3:** Cohorts 1 and 2 Kiosk interaction and average daily student playtime frequency and duration

					Count of active kiosk days with kiosk average daily play time >50, 100, and 150 s					
					>50 s		>100 s		>150 s	
Site	Start date	End date	Intervention duration days	Active kiosk days	*n*	%	*n*	%	*n*	%
*Cohort 1*										
School 1	August 29, 2018	April 16, 2019	230	60 (26.1%)	38	63.3	20	33.3	4	6.7
School 2	August 30, 2018	March 27, 2019	209	65 (31.1%)	52	80.0	16	24.6	8	12.3
School 3	September 6, 2018	April 29, 2019	235	69 (29.4%)	62	89.9	30	43.5	7	10.1
School 4	September 11, 2018	March 28, 2019	198	24 (12.1%)	15	62.5	1	4.2	0	0
School 5	September 17, 2018	March 29, 2019	193	52 (26.9%)	36	69.2	0	0.0	0	0
School 6	September 18, 2018	April 30, 2019	224	39 (17.4%)	15	38.5	5	12.8	0	0
*Cohort 2*										
School 7	September 17, 2019	March 6, 2020	171	88 (51.5%)	76	86.4	62	70.5	37	42.0
School 8	September 19, 2019	January 29, 2020	132	25 (18.9%)	23	92.0	19	76.0	14	56.0
School 9	September 24, 2019	March 3, 2020	161	64 (39.8%)	64	100.0	56	87.5	39	60.9
School 10	September 26, 2019	March 6, 2020	162	41 (25.3%)	38	92.7	31	75.6	28	68.3

Results indicated variability in the duration of kiosk accessibility as school 4 had 24 days when the kiosk was made available to children, and schools 2 and 3 had almost three times the number of days of kiosk exposure. At low-exposure schools (schools 4 and 6), playtime duration was also low, as evidenced by the proportion of days exceeding the three cutoff levels of playtime. For example, school 6 had only 12.8% of 39 days that exceeded 100 s of daily playtime (*n* = 5 days). At the cutoff level of >100 s of playtime, schools 4 and 5 had effectively no engagement with the kiosk. Cohort 2 schools had substantively higher levels of overall adherence, where kiosks were available on 34.8% of possible days compared with the 24.0% of days for cohort 1. Child playtime days >100 s were greater than 70% in cohort 2, and days with playtime greater than 150 s were between 42% and 68% of active kiosk days.

## Discussion

Despite the well-documented benefits of PA, research indicates a global trend of significant decline in PA over the past two decades^31^. The decline of MVPA in children, in particular, accelerates over time and begins a precipitous drop around when children reach 9–11 years of age, with recent meta-analyses noting that the downhill trend is worse for girls than for boys^32^. In response to this age-related decline in PA, childhood before adolescence has been identified as a window of opportunity for PA interventions^9^. Due to the historical emphasis on MVPA (vs. light intensity PA) in adolescents, detailed tracking and analyses of longitudinal PA at varying intensity levels and sedentary behavior changes in pre-adolescent children are scarce^33,34^; the current findings presented in this study address this gap in the research by tracking 303 children’s PA over six months across 19 different study sites.

Earlier findings have noted that schools and afterschool programs may serve as environments conducive to children’s PA interventions^35^, parental support is a predictor of child PA success^36^, and designing interventions that promote enjoyable PA is important for younger children^37^. Guided by self-determination theory^38^, this study presents a large-scale randomized clinical trial to leverage emerging technologies in a cost- and labor-effective precision health system to promote individually tailored PA interventions over a 6-month intervention for pre-adolescent children (*N* = 303) in 19 afterschool sites.

The VFB ecosystem was designed to combine the power of wearables and sensors that allow unobtrusive, objective, and consistent tracking of PA with smartphones that keep parents involved with their child’s participation and a mixed reality virtual agent programmed to guide children in autonomously determining the pace of the intervention and to provide motivational support. Current findings support the potential effectiveness of the VFB ecosystem in promoting beneficial changes in children’s PA and sedentary behavior. The ecosystem significantly increased PA and reduced sedentary behavior over the short term and then was successful at sustaining baseline levels of PA and sedentary behavior as opposed to the precipitous pre-adolescent drop in PA and increase in sedentary behavior commonly observed in this age group of children. Posthoc analyses implied that these effects may be more pronounced for children who own dogs than children who are not dog owners, suggesting that children with dogs may have perceived the VFB ecosystem as an extension of the human-pet relationship in their physical world.

Early movement guidelines for children and adolescents focused primarily on MVPA and, later, sedentary behavior. Recent youth guidelines have adopted a 24-h integrated movement perspective, including PA of all intensities, sedentary behavior, and sleep^39^. The impetus for this change has been driven by a growing body of evidence that (1) each of these behaviors can significantly impact physical and mental health in youth, (2) these behaviors may interact with each other to influence health outcomes, and (3) changing the amount of time spent in one movement behavior will inevitably influence time spent in one or more of the other 24-h movement behaviors^40^. The current intervention was particularly effective in converting significant levels of sedentary behavior with light-intensity PA, with children who interacted with the ecosystem for three months (Time 2) engaging in significantly higher levels of light-intensity PA than children in the active control group who did not receive support from their parents or from the virtual pet. Similar trends of activity were observed at Time 2 for MVPA, although the treatment effect was not significant compared to the control condition.

Our findings suggest that the VFB ecosystem was successful in converting significant levels of sedentary behavior with light-intensity PA for up to three months. However, the current design of the VFB ecosystem seemed to be insufficient in overcoming the downward trend of PA observed between Time 2 and Time 3. The drop-off in PA after three months may be due to seasonality, wherein earlier studies have confirmed significantly lower PA and higher sedentary behavior in children during the winter months (months 3–6 of the current intervention)^41^, or lack of sufficiently motivating content during the digital interactions with the virtual pet, wherein digital content must be updated to provide new challenges to users who have mastered existing content to sustain enjoyment and engagement with the ecosystem^42,43^. Access to the kiosk and the virtual dog was also tied to the afterschool calendar; when the afterschool was closed (e.g., holidays), children could not access the kiosk or interact with the virtual dog. Longer observation periods at school sites over multiple academic years may be needed to differentiate cohort and seasonality effects from ecosystem implementation effects.

The sensitivity analyses point to the importance of design considerations for PA interventions targeting children by demonstrating that greater compliance with the intervention protocol leads to greater treatment effects^28^. Design enhancements in technology-mediated health interventions can minimize staff involvement and increase user compliance; technology integrated into the system should be largely self-contained to reduce user input, data tracking should be unobtrusive, and regular reminders should be integrated to maintain user engagement. Earlier research has found that digital health interventions with customizable features lower user attrition^44^ and increase user engagement, particularly for people who have a high need for autonomy^45^. Current findings also add support to the strength of precision health approaches that customize interventions through digital technologies, such as algorithm-driven virtual agents, wearables, and sensors.

Emerging research notes that light-intensity PA through free-form, unstructured play served is a major source of children’s PA, and MVPA makes only minor contributions to unstructured energy expenditure^46^. Increasing total PA through unstructured play assisted via the VFB ecosystem offers a practical and potentially sustainable solution for children by supporting their PA efforts through encouragement from parents and the virtual agent, providing accurate and consistent feedback, and emphasizing the intrinsic enjoyment of PA. With the continuing popularity and accessibility of wearable and sensor technologies, the current findings indicate that integrating the power of technologies in PA interventions can help children serve as autonomous vehicles of their own behavior change. These findings may also inform the design of games that were not intended to serve as a health intervention (e.g., Pokemon Go) that can indirectly contribute to increasing children’s unstructured play^47^.

At scale, the VFB ecosystem is anticipated to maximize public health benefits for participating children and allow organizations to reinvest resource savings into other public health priorities. The VFB ecosystem represents a new generation of technology-mediated precision health interventions for children, which integrates fun and engaging technology into existing social systems to promote sustainable lifestyle changes. Because the ecosystem is a cost- and labor-efficient solution that integrates consumer-grade technology with low barriers for continued use, it has the potential for rapid diffusion and widespread public health impact.

## Methods

### Study design and participants

**Fig. 3:**
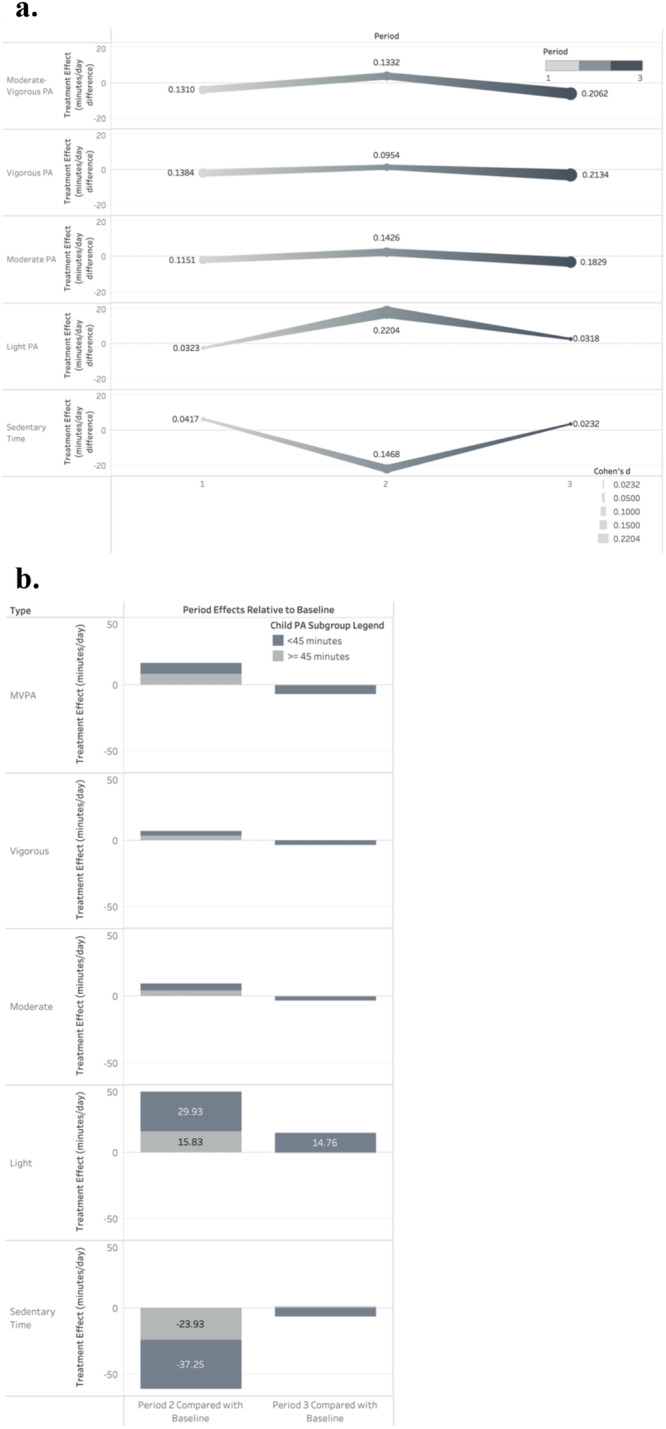
Standardized treatment effects and stratified pa and sedentary time by children less than 45 min and at least 45 min of MVPA at baseline. Plots of the effect size Cohen’s *d* of randomization to the VFB intervention are used to demonstrate patterns of PA and sedentary time over the course of three periods of intervention and by subpopulation MVPA stratum. The *x*-axis corresponds to the time period in 3-month intervals, and the *y*-axis is the standardized treatment effect size. When the bar is below a value of zero, the outcome was observed to be smaller in the intervention arm compared to the control arm (e.g., sedentary time). Down rows of each figure are each PA and sedentary time outcome observed over the intervention. Where effect size Cohen’s *d* is not presented, the *y*-axis represents the growth or decline in treatment effects for each period in time compared with the baseline period within each stratum of children with less than 45 min and at least 45 min of MVPA at baseline. **a** At 3-month follow-up, sedentary time was most strongly converted to light-intensity PA, and the effect reverted to approximately the baseline level by 6-month follow-up. **b** Children with under 45-min of PA at baseline had the largest decline in sedentary time between baseline and 3-month follow-up.

All procedures for this multisite, cluster-randomized, controlled trial were approved by the University of Georgia Institutional Review Board and pre-registered on ClinicalTrials.gov (NCT03524183). Two-hundred fifty-seven children were recruited for cohort 1, and 165 children were recruited for cohort 2 (Fig. 3). The study was advertised to the YMCA afterschool program families by posting flyers on the YMCA afterschool websites and at YMCA facilities, sending detailed letters to parents, emailing information to parents, and having researchers visit each facility to distribute materials and speak with families. Researchers then contacted interested families to screen for eligibility.

Child and parent eligibility was based on (a) having at least one child in grades 1–5 (between the ages of 6–11) enrolled in the afterschool program (b) participating in moderate-intensity PA without assistance, and (c) attending the afterschool program most days of each week throughout the school year. Upon being screened eligible, parent and child dyads attended in-person orientations to learn about the study and its requirements for participation. If still interested in participating, parents and children signed informed consent and assent forms, respectively, and were then recorded as official participants. The study protocol is available online.

### Randomization and masking

Because children interact with one another in after-school program activities and can observe others in the same school/branch assigned to treatments differing from their own assignment, a cluster-randomized design was used to assign treatments at the after-school program level. To control for the potential impact of socio-economic status on the study outcomes, we randomized after-school programs to VFB and control treatments in matched pairs, matched by the percentage of children in those programs receiving free lunches.

The study was non-masked for all participants, YMCA staff, and the research team. Children and parents were not informed about the results of the randomization, but the interventions carried out at each of the afterschool sites were not masked at any point of the study. YMCA staff were also not informed about their random assignment to treatment or control groups. The allocation variable for the randomization result was added into the dataset at the time of analysis after cleaning the data.

### Procedures

The intervention began at baseline (Time 1), during which researchers set up the motion-tracking kiosks at each afterschool site and trained YMCA staff. At Time 1, children in the treatment condition were introduced to the kiosk and shown how to use the VFB system, how to set up their unique, customized virtual dog, and how to interact with it. Playtime with the pet was designed as a single-user experience that allowed the child to set up PA goals, receive accurate feedback and social support, and increase perceived relatedness between the child and the pet. Children in the control condition were shown how to use the computer system for setting PA goals. Children were then assessed for body composition including height, weight, and waist circumference, and completed baseline surveys. All children received Fitbits.

To validate the PA measured through the Fitbit (validation results reported elsewhere^48^), at each of the three measurement periods, up to 20 children at each after-school program were fitted with ActiGraph GT9X accelerometers attached to an elastic belt and positioned at the mid-axillary line of the right hip. Children were instructed to wear the device during all waking hours, except for water-based activities, for seven continuous days. Parents recorded daily wear times and reasons for non-wear. At 3 months post-baseline (Time 2) and 6 months post-baseline (Time 3), surveys and body composition measurements were again administered, and Actigraphs were distributed and subsequently returned by mail after 1 week.

For children in the treatment condition, in addition to collecting daily PA data from children, the Fitbit data activated real-time updates to the virtual environment and the virtual dog. When a child’s Fitbit data synced with the kiosk, that child’s virtual dog became fitter and happier (as rendered through appearance and behavior) in the kiosk. The kiosk was intentionally designed to discourage children from engaging in extended PA in front of it. Children were able to play with the dog for a limited amount of time (~10 min) when they met their self-determined PA goal. Because the ecosystem was designed to encourage and support children’s PA, the virtual dog did not become unhealthy; rather, the dog provided accurate feedback and encouraged the child to leave the kiosk to engage in further PA. Parents received real-time updates about the child’s interactions with the kiosk via their smartphone, and the virtual dog tailored its response based on the child’s PA performance, guiding and supporting the child through PA performance reviews and new PA goal setup. The kiosks were retrieved from the treatment sites at the end of Time 3. Between cohorts 1 and 2, some system enhancements were made to the kiosk to enhance its usability^28^. The changes to the user interface allowed children to better sync their Fitbit data with the kiosk and made the login process easier; no changes were made to the virtual dog itself to ensure consistency of treatment between the two cohorts.

### Outcomes

The primary outcome of the impact was children’s physical activity and sedentary behavior. ActiGraph devices were equipped with Firmware v1.7.1 and Actilife software version 6.13.4 was used to initialize and download data in 10-s epochs. Accelerometer counts were used to classify each epoch into a PA intensity category (i.e., sedentary, light, moderate, vigorous) using the age-appropriate cut-points developed by Evenson et al.^49^ Device wear time was estimated using the Choi algorithm^50^, where periods with zero count values for 90 consecutive minutes or longer were classified as non-wear. Intensity estimates, as well as steps, were summed for each valid day of wear (days with 8+ hours of ActiGraph wear). ActiGraph reliability and validity have been consistently demonstrated^51^, and it has been shown to correlate reasonably with activity energy expenditure measured by doubly labeled water^52^.

Playtime (i.e., time durations when the child was actively playing with the pet through the kiosk) was calculated as the total daily time a child interacted with the virtual dog through the kiosk. Play was designed to increase vicarious reinforcement and self-efficacy; the more PA in the physical world the child obtained (as tracked through Fitbit), the greater the stamina that the child’s virtual dog would gain, and the longer the dog would be able to interact with the child.

Although the protocol had planned for both Cohort 1 and 2 to collect data for the school year, the COVID-19 global pandemic shut down most of the afterschool programs after Time 3 of Cohort 2. Therefore, the analyses reported here reflect data collected from the first six months of both cohorts (Time 1, Time 2, Time 3). No adverse events were monitored actively, and none were reported by YMCA staff or researchers during the intervention.

### Statistical analysis

Descriptive statistics and frequency tabulations were computed to examine the balance of socio-demographic characteristics at the individual and household levels between the treatment (T) and control (C) groups for exchangeability. Cohorts 1 and 2 were pooled for intent-to-treat analysis (ITT) and separately stratified to examine the interaction of modified study protocols implemented in cohort 2 in a sensitivity analysis. The same procedure was performed to examine whether children with lower baseline MVPA (>45 min) responded more favorably to the intervention compared with the children with at least 45 minutes of baseline MVPA. A final sensitivity analysis was conducted to examine whether children who currently had a pet dog (about one-third of the sample) responded more favorably to the intervention compared with those who did not currently own a dog.

The primary outcome of interest was the interaction of period (baseline, 3-month follow-up, 6-month follow-up) with the treatment group, each treated as a categorical random variable, to examine non-linear treatment group period differences in child physical activity (PA) and sedentary behavior (SB) over the three study periods (Time 1, Time 2, Time 3). Child PA and sedentary behavior were measured in average minutes per day except for daily steps, and all were treated as continuous outcomes.

Although randomization exhibited an overall balance of demographics, dog ownership, and anthropometric factors between treatment and control groups, model adjustment for child accelerometer wear time and overweight status (sex-age adjusted BMI%ile≥85), child sex, race, and age, and the educational attainment and overweight status of the primary caregiver were included to reduce unexplained variation in the models to improve precision. Hierarchical linear mixed models were fitted to the longitudinal accelerometer data and accounted for correlated PA and sedentary behavior error terms using a random intercept for school and a random intercept for children nested within schools. A random slope for days from baseline was fitted to account for child-level PA and sedentary behavior daily trajectories over the three stages of the intervention. School-level and child-level intraclass-correlation coefficients (ICCs) were used to characterize the correlation of outcomes within the two levels of interest. All models include Huber-White, cluster-correlated robust standard errors, and two-sided, a priori statistical significance was set to the 0.05 level for all inferential procedures. Data management and analysis were conducted in Stata 17.0 MP. The trial is registered at ClinicalTrials.gov, NCT03524183, and is active but not recruiting.

### Reporting summary

Further information on research design is available in the Nature Research Reporting Summary linked to this article.

### Supplementary information

Reporting Summary

## Data Availability

The datasets generated during and/or analyzed during the current study are available from the corresponding author upon reasonable request.
