# Supplementary material for: Virtual fitness buddy ecosystem: a mixed reality precision health physical activity intervention for children
Source: NPJ Digit Med. 2024 May 21;7:134. doi: 10.1038/s41746-024-01133-5 (PMC11109260; doi:10.1038/s41746-024-01133-5)
Supplement: Supplementary file 1 — Reporting Summary [file 41746_2024_1133_MOESM1_ESM.pdf]

nature portfolio

Corresponding author(s): Sun Joo Ahn

Last updated by author(s): Mar 8, 2024

Reporting Summary

Nature Portfolio wishes to improve the reproducibility of the work that we publish. This form provides structure for consistency and transparency in reporting. For further information on Nature Portfolio policies, see our Editorial Policies and the Editorial Policy Checklist.

Statistics

For all statistical analyses, confirm that the following items are present in the figure legend, table legend, main text, or Methods section.

n/a	Confirmed	
☐	☒	The exact sample size (*n*) for each experimental group/condition, given as a discrete number and unit of measurement
☐	☒	A statement on whether measurements were taken from distinct samples or whether the same sample was measured repeatedly
☐	☒	The statistical test(s) used AND whether they are one- or two-sided<br>*Only common tests should be described solely by name; describe more complex techniques in the Methods section.*
☐	☒	A description of all covariates tested
☐	☒	A description of any assumptions or corrections, such as tests of normality and adjustment for multiple comparisons
☐	☒	A full description of the statistical parameters including central tendency (e.g. means) or other basic estimates (e.g. regression coefficient) AND variation (e.g. standard deviation) or associated estimates of uncertainty (e.g. confidence intervals)
☐	☒	For null hypothesis testing, the test statistic (e.g. *F*, *t*, *r*) with confidence intervals, effect sizes, degrees of freedom and *P* value noted<br>*Give P values as exact values whenever suitable.*
☒	☐	For Bayesian analysis, information on the choice of priors and Markov chain Monte Carlo settings
☐	☒	For hierarchical and complex designs, identification of the appropriate level for tests and full reporting of outcomes
☐	☒	Estimates of effect sizes (e.g. Cohen's *d*, Pearson's *r*), indicating how they were calculated

Our web collection on statistics for biologists contains articles on many of the points above.

Software and code

Policy information about availability of computer code

Data collection	ActiGraph devices were equipped with Firmware v1.7.1 and Actilife software version 6.13.4 was used to initialize and download data in 10-second epochs
Data analysis	Accelerometer counts were used to classify each epoch into a PA intensity category (i.e., sedentary, light, moderate, vigorous) using the age-appropriate cut-points developed by Evenson et al. Device wear time was estimated using the Choi algorithm. Data management and analysis was conducted in Stata 17.0 MP.

For manuscripts utilizing custom algorithms or software that are central to the research but not yet described in published literature, software must be made available to editors and reviewers. We strongly encourage code deposition in a community repository (e.g. GitHub). See the Nature Portfolio guidelines for submitting code & software for further information.

Data

Policy information about availability of data

All manuscripts must include a data availability statement. This statement should provide the following information, where applicable:

- Accession codes, unique identifiers, or web links for publicly available datasets
- A description of any restrictions on data availability
- For clinical datasets or third party data, please ensure that the statement adheres to our policy

The datasets generated during and/or analyzed during the current study are available from the corresponding author on reasonable request.

Research involving human participants, their data, or biological material

Policy information about studies with human participants or human data. See also policy information about sex, gender (identity/presentation), and sexual orientation and race, ethnicity and racism.

Reporting on sex and gender	Findings reported in the current manuscript do not apply to only one sex or gender. Sex and gender were not considered in the study design as the physical activity intervention was designed as a primary prevention program targeting both boys and girls. Sex and gender of the sample were determined based on self-reporting.
Reporting on race, ethnicity, or other socially relevant groupings	Demographic variables, including race, ethnicity, and income were collected for the clinical trial to determine successful randomization across the two arms (Table 1); however, they were not used in the analysis as the study design did not specifically target a race, ethnic group, or other socially relevant grouping. The demographic information was collected through self-report. We did not use any of these variables as proxy for other socially constructed/relevant variables.
Population characteristics	See the Behavioural and Social Sciences Study Design questions below.
Recruitment	Study participants were recruited in collaboration with the YMCA. The study was advertised to the YMCA afterschool program families by posting flyers on the YMCA afterschool websites and at YMCA facilities, sending detailed letters to parents, emailing information to parents, and having researchers visit each facility to distribute materials and speak with families. Researchers then contacted interested families to screen for eligibility. It is unlikely that biases impacted the recruitment process.
Ethics oversight	All procedures for this multisite, cluster randomized, controlled trial was approved by University of Georgia Institutional Review Board

Note that full information on the approval of the study protocol must also be provided in the manuscript.

Field-specific reporting

Please select the one below that is the best fit for your research. If you are not sure, read the appropriate sections before making your selection.

☐ Life sciences ☒ Behavioural & social sciences ☐ Ecological, evolutionary & environmental sciences

For a reference copy of the document with all sections, see nature.com/documents/nr-reporting-summary-flat.pdf

Behavioural & social sciences study design

All studies must disclose on these points even when the disclosure is negative.

Study description	The Virtual Fitness Buddy (VFB) ecosystem is a community-level precision health intervention for children that integrates the theoretical tenets into a scalable and cost-effective PA program to increase and sustain PA in children through unstructured play. We tested the efficacy of the VFB ecosystem across two cohorts of parent-child dyads recruited from 19 elementary schools and YMCA branches, each lasting for six months during the academic year. We compared the VFB ecosystem's efficacy in increasing PA among 6-11 year-old children against an active control group, which employed a computer system to assist children in setting and meeting PA goals but without the social support or feedback from the virtual dog or their parents.
Research sample	Two-hundred fifty-seven children (age 6-11)were recruited for cohort 1, and 165 children (age 6-11) were recruited for cohort 2
Sampling strategy	Child and parent eligibility was based on (a) having at least one child in grades 1-5 (between the ages of 6–11) enrolled in the afterschool program who (b) could participate in moderate intensity PA without assistance and who (c) attended the afterschool program most days of each week throughout the school year. Upon being screened eligible, parent and child dyads attended in-person orientations to learn about the study and its requirements for participation. If still interested in participating, parents and children signed informed consent and assent forms, respectively, and were then recorded as official participants.
Data collection	The primary outcome of impact was children's physical activity and sedentary behavior. ActiGraph devices were equipped with Firmware v1.7.1 and Actilife software version 6.13.4 was used to initialize and download data in 10-second epochs. Accelerometer counts were used to classify each epoch into a PA intensity category (i.e., sedentary, light, moderate, vigorous) using the age-appropriate cut-points developed by Evenson et al. Device wear time was estimated using the Choi algorithm, where periods with zero count values for 90 consecutive minutes or longer were classified as non-wear. Intensity estimates, as well as steps, were summed for each valid day of wear (days with 8+ hours of ActiGraph wear).
Timing	Although the protocol had planned for both Cohorts 1 and 2 to collect data for the school year, the COVID-19 global pandemic shut down most of the afterschool programs after Time 3 of Cohort 2. Therefore, the analyses reported here reflect data collected from the first six months of both cohorts (Time 1, Time 2, Time 3).
Data exclusions	No adverse events were monitored actively and none were reported by YMCA staff or researchers during the intervention.
Non-participation	No adverse events were monitored actively and none were reported by YMCA staff or researchers during the intervention.
Randomization	Because children interact with one another in after school program activities and can observe others in the same school/branch

Randomization	assigned to treatments differing from their own assignment, a cluster-randomized design was used to assign treatments at the after-school program level. To control for the potential impact of socio-economic status on the study outcomes, we randomized after school programs to VFB and control treatments in matched pairs, matched by the percentage of children in those programs receiving free lunches.

Reporting for specific materials, systems and methods

We require information from authors about some types of materials, experimental systems and methods used in many studies. Here, indicate whether each material, system or method listed is relevant to your study. If you are not sure if a list item applies to your research, read the appropriate section before selecting a response.

Materials & experimental systems

n/a	Involved in the study	
☒	☐	Antibodies
☒	☐	Eukaryotic cell lines
☒	☐	Palaeontology and archaeology
☒	☐	Animals and other organisms
☐	☒	Clinical data
☒	☐	Dual use research of concern
☒	☐	Plants

Methods

n/a	Involved in the study	
☒	☐	ChIP-seq
☒	☐	Flow cytometry
☒	☐	MRI-based neuroimaging

Clinical data

Policy information about clinical studies

All manuscripts should comply with the ICMJE guidelines for publication of clinical research and a completed CONSORT checklist must be included with all submissions.

Clinical trial registration	NCT03524183
Study protocol	Online supplementary file
Data collection	We tested the efficacy of the VFB ecosystem across two cohorts of parent-child dyads recruited from 19 elementary schools and YMCA branches in the metro-Atlanta, Georgia area, each lasting for six months during the academic year. Date of first enrollment was August 2018.
Outcomes	The primary outcome of impact was children's physical activity and sedentary behavior. ActiGraph devices were equipped with Firmware v1.7.1 and Actilife software version 6.13.4 was used to initialize and download data in 10-second epochs. Accelerometer counts were used to classify each epoch into a PA intensity category (i.e., sedentary, light, moderate, vigorous) using the age-appropriate cut-points developed by Evenson et al.

Plants

Seed stocks	*Report on the source of all seed stocks or other plant material used. If applicable, state the seed stock centre and catalogue number. If plant specimens were collected from the field, describe the collection location, date and sampling procedures.*
Novel plant genotypes	*Describe the methods by which all novel plant genotypes were produced. This includes those generated by transgenic approaches, gene editing, chemical/radiation-based mutagenesis and hybridization. For transgenic lines, describe the transformation method, the number of independent lines analyzed and the generation upon which experiments were performed. For gene-edited lines, describe the editor used, the endogenous sequence targeted for editing, the targeting guide RNA sequence (if applicable) and how the editor was applied.*
Authentication	*Describe any authentication procedures for each seed stock used or novel genotype generated. Describe any experiments used to assess the effect of a mutation and, where applicable, how potential secondary effects (e.g. second site T-DNA insertions, mosiacism, off-target gene editing) were examined.*
